# Phytochemicals with protective effects against acute pancreatitis: a review of recent literature

**DOI:** 10.1080/13880209.2022.2039723

**Published:** 2022-02-18

**Authors:** Yao Tang, Mingli Sun, Zhenning Liu

**Affiliations:** a Department of Emergency Medicine, Shengjing Hospital of China Medical University, Shenyang, China; b School of Kinesiology, Shenyang Sport University, Shenyang, China

**Keywords:** Inflammation, oxidation, signalling pathway, molecular mechanism, chemicals, pancreatic damage

## Abstract

**Context:**

Acute pancreatitis (AP) is an acute abdominal inflammatory disease with episodes ranging from mild to fulminant symptoms which could include necrosis, systemic inflammation and multiple organ dysfunction. Increasing experimental evidence demonstrates that specific bioactive ingredients from natural plants have a favourable therapeutic effect on AP.

**Objective:**

The objective of this review is to summarize the protective effects and potential mechanisms of action of phytochemicals on the attenuation of AP.

**Methods:**

Experimental studies *in vivo* or *in vitro* between January 2016 and June 2021 were sought in PubMed and Web of Science using the following search terms: (‘phytochemicals’ OR ‘medicinal plant’ OR ‘traditional medicine’) AND (‘pancreatitis’ OR ‘pancreatic damage’ OR ‘pancreatic injury’). Data concerning the basic characteristics of phytochemicals, therapeutic dose and potential molecular mechanisms related to AP were extracted in this study.

**Results:**

A total of 30 phytochemicals with potential therapeutic effects were reviewed and summarized systematically. According to their molecular pathways in AP, the underlying mechanisms of the phytochemicals were illustrated in detail.

**Discussion and conclusions:**

The phytochemicals with anti-inflammatory and antioxidant abilities may be efficient candidate drugs for AP treatment. Importantly, more preclinical investigations are needed to illustrate the efficacy of future phytochemicals.

## Introduction

Acute pancreatitis (AP) is one of the most common digestive disorders worldwide (Karakayali 2014). Unfortunately, approximately 20% of these patients develop severe life-threatening pancreatitis requiring intensive care (Tenner et al. 2013; Zhang et al. 2020). The incidence of AP is still increasing annually. Accumulating evidence implicates alcohol, gallstone disease, metabolic abnormalities and obesity as the predominant causes of AP. Complex intra-acinar events, including zymogen activation, autophagy, oxidative stress, mitochondrial dysfunction and endoplasmic stress, have been reported to be involved in the pathogenesis of AP (Jiang et al. 2020). In addition, the development of AP is partly attributed to the inflammatory response which involves recruitment of immune cells, activation of damage-associated molecular patterns (DAMPs) and release of various inflammatory cytokines and chemokines.

In the past two decades, although life-supporting medical device applications have improved the prognosis of severe AP to some extent, it is generally accepted that some synthetic drugs are greatly beneficial for the treatment of AP. Many traditional Chinese medicines have been widely used to treat AP in China (Li et al. 2017). Some traditional Chinese medicines including Chaiqin chengqi decoction (Wen et al. 2020), Dachengqi decoction (Sun et al. 2020), Da-Chai-Hu decoction (Zhao G et al. 2019) and Qingyi decoction (Su et al. 2019), exert therapeutic effects on AP. Although these research reports on the decoctions mentioned above are mainly from Eastern Asian countries, these studies show that these decoctions contribute to the improvement of the prognosis of patients with AP. What are thought to be the most important ingredients of the decoctions mentioned above are mostly from natural plants. Based on previous reports, many compounds from natural plants have been studied to investigate their ability to attenuate pancreatic cell damage *in vivo* or *in vitro*.

This review provides a reference for future drug discovery in the treatment of AP. Therefore, we did not focus on the drugs thought to affect AP outcomes from clinical practice reports, but we systematically reviewed previous experimental studies between January 2016 and June 2021 investigating phytochemicals. We have comprehensively summarized the protective effects and the proposed underlying mechanisms of phytochemicals against AP. This review will not only update the understanding of phytochemicals in the treatment of AP, but also discuss the protective effects and molecular mechanisms of the plant-based compounds.

## Acute pancreatitis models and the major signalling pathways

Acute pancreatitis is a complex disease caused by various pathogenic factors including biliary tract disease, excessive alcohol consumption, hyperlipidaemia, etc. As for the experimental studies, many unrelated stimuli were used to replicate human AP according to the previously published literature. Sodium taurocholate (NaT) or cerulein was mostly used to establish an experimental animal model of AP. NaT was retrogradely injected into the pancreatic duct to cause pancreatic acinar cells damage, and cerulein could promote the secretion of pancreatic proteolytic enzymes, leading to trypsinogen activation in pancreatic acinar cells. In addition, as shown in Table 1, hyperlipidaemia induced by a high‑fat diet or l-arginine injection in mice or rats was also used to induce AP. Nevertheless, none of the animal models can fully simulate the pathology of AP in humans.

**Table 1.** Phytochemicals and the potential molecular mechanism.

Phytochemicals	Origins	Therapeutic dose	Animals/cell	Inducers of AP model	Molecular mechanism	Reference
*Anti-inflammation*						
α/β-ABA	*Boswellia sacra* Flückiger-Dupiron	100 mg/kg (p.o.); 2 μM *in vitro*	Male C57BL/6 mice; RAW264.7 cells	Cerulein	Downregulate MAPKs pathway	Zhang et al. (2020)
Artesunate	*Artemisia annua* L.	3.50 mg/kg (i.m.)	Male SD rats	NaT	Inhibit TLR4/NF-κB pathway	Cen et al. (2016)
Baicalein	*Scutellaria baicalensis* Georgi	20 mg/kg (i.v.); 10 µM *in vitro*	C57BL/6 mice; RAW264.7 cells	Cerulein	Inhibit NF-κB, MAPK and STAT3 pathways	Pu et al. (2019)
Baicalin	*Scutellaria baicalensis* Georgi	20 mg/kg (i.v.); 40 µM *in vitro*	C57BL/6 mice; AR42J cells	Cerulein	Increase miR-15a and inhibit MAP2K4/JNK pathways	Zhen et al. (2021)
		200 mg/kg (i.v.)	SD rats	NaT	Downregulate PKD1 and NF-κB pathway	Qian et al. (2018)
Berberine	*Coptis chinensis* Franch	20 mg/kg (i.p.)	Female C57BL/6 mice	CDE	Inhibit JNKs, p38 MAPK and NF-κB	Choi et al. (2017)
Chlorogenic acid	*Lonicera japonica* Thunb.	40 mg/kg (i.p.)	Male C57BL/6 mice	l-Arginine	Downregulate inflammatory cytokine IL-6, MIP-2 and MIF	Ohkawara et al. (2017)
Oxymatrine	*Sophora alopecuroides* L.	250 mg/100 g (i.p.)	Male Wistar rats	l-Arginine	Inhibit MAPK/NF-κB pathway	Zhang Z et al. (2019)
Quercetin	Fruits and vegetables	40 mg/kg (i.v.); 40 µM *in vitro*	C57BL/6 mice; AR42J cells	Cerulein	Inhibit p38/MAPK pathway by upregulating miR-216b	Sheng et al. (2021)
Saikosaponin a	*Bupleurum chinense* DC.	20 mg/kg (i.p.)	Male SD rats	Hyperlipidemic	Suppress NF-κB pathway and promote PPAR-γ expression	Feng et al. (2019)
Tetramethylpyrazine/ligustrazine	*Oreocome striata* (Wall. ex DC.) Pimenov & Kljuykov	150 mg/kg (i.p.); 7.35 mM *in vitro*	SD rats; AR42J cells	Cerulein	Suppress p38 and ERK/MAPK pathways; increase the levels of p53 and cleaved caspase 3	Chen et al. (2016)
		10 mg/kg (i.p.)	C57BL/6	Cerulein	Inhibit NF-κB pathway	Chen et al. (2019)
Visnagin	*Ammi visnaga* (L.) Lam.	60 mg/kg (i.p.)	Male Swiss albino mice	Cerulein	Improve Nrf2 expression and suppress NF-κB pathway	Pasari et al. (2019)
Withaferin A	*Withania somnifera* (L.) Dun.	10 mg/kg (i.p.)	Male Swiss mice	Cerulein	Upregulate Nrf2, inhibit NF-κB and increase Bcl-2	Tiruveedi et al. (2018)
*Antioxidation*						
Carvacrol	*Oreganum vulgare* Linn.	200 mg/kg (i.p.)	Male SD rats	Cerulein	Increase SOD, catalase and GSH-Px; reduce MDA	Kilic et al. (2016)
Dihydrokaempferol	*Bauhinia championii* (Benth.) Benth.	80 mg/kg (p.o.); 50 µM *in vitro*	C57BL/6 mice; mice primary acinar cells	Cerulein with LPS	Inhibit Keap1 and promote Nrf2 pathway	Liang et al. (2020)
Isoliquiritigenin	*Glycine max (L.)* Merr. *Glycyrrhiza glabra* L.*, Allium ascalonicum* L.	200 mg/kg (i.p.)	Male ICR mice	Cerulein	Increase Nrf2/HO-1 pathway	Liu et al. (2018)
Rutin	Citric fruits and grapes	150 mg/kg (p.o.)	Male Swiss mice	l-Arginine	Diminish 3-nitrotyrosine and MPO; increase SOD and catalase	Abreu et al. (2016)
Tanshinone IIA	*Salvia miltiorrhiza* Bunge	25 mg/kg (i.p.)	Male ICR mice	Cerulein	Upregulate Nrf2/ROS pathway	Chen et al. (2020)
Compound from *Coreopsis tinctoria* Nutt.	*Coreopsis tinctoria* Nutt.	12.5 mg/kg (8.7 mg/kg) (i.p.); 0.5 mM *in vitro*	Male Balb/c mice; male C57/BL6J mice (male Wistar rats)	Cerulein; TLCS; NaT	Activate Nrf2 pathway	Du et al. (2018)
Compound 1 from *Dioscorea zingiberensis*	*Dioscorea zingiberensis* C. H. Wright	0.5 mM *in vitro*	Pancreatic acinar cells	NaT	Decrease ATP depletion and ROS generation	Du et al. (2017)
Compound 2 from *Dioscorea zingiberensis*	*Dioscorea zingiberensis* C. H. Wright	2.5 mM *in vitro*	Cells from Balb/c mice	NaT	Increase ATP and decrease ROS	Zhang et al. (2016)
*Anti-inflammation and antioxidation*						
Borneol	*Acorus calamus* L.	300 mg/kg (p.o.)	Male Swiss albino mice	Cerulein	Regulate Nrf2/NF-κB pathway	Bansod et al. (2021)
Calycosin	*Astragalus membranaceus* (Fisch.) Bunge	50 mg/kg (i.p.)	Male Balb/C mice	Cerulein	Inhibit p38 MAPK and NF-κB pathway; increase the SOD activity	Ma et al. (2018)
Curcumin	*Curcuma longa* L.	50 and 200 mg/kg (i.p.)	Male ICR mice	l-Arginine	Inhibit TLR-4/NF-κB and p38 MAPK signal pathway	Siriviriyakul et al. (2019)
		200 mg/kg (i.p.); 27.17 µM *in vitro*	Female SD rats; AR42J cells	NaT; cerulein with LPS	Inhibit p38 MAPK pathway	Wang et al. (2019)
Ellagic acid	Fruits and vegetables	85 mg/kg (p.o.)	Male SD rats	l-Arginine	Reduce TNF-α, IL-1β and IL-6; increase the total antioxidant capacity	Yılmaz et al. (2016)
Emodin	*Rheum rhabarbarum* L.	60 mg/kg (i.g.)	Male SD rats	NaT	Inhibit P2X7/NLRP3 pathway	Zhang Q et al. (2019)
		40 µM *in vitro*	AR42J cells	Cerulein with LPS	Inhibit ROS-mediated activation of JNK and p38/MAPK pathway	Zhao et al. (2019)
Luteolin	*Reseda odorata* L.	100 mg/kg (i.p.)	Male ICR rats	Cerulein with LPS	Activate HO-1 and Inhibit NF-κB pathway	Xiong et al. (2017)
Picroside II	*Picrorhiza scrophulariiflora* Pennell	25 mg/kg (i.v.)	Male SD rats	Cerulein	Inhibit NF-κB pathway	Piao et al. (2017)
Resveratrol	*Polygonum cuspidatum* Siebold & Zucc.	50 mg/kg (p.o.)	SD rats	Cerulein	Inhibit PI3Ks/Akt and NF-κB pathway; activate Nrf2	Tsang et al. (2016); Agah et al. (2021)
		80 mg/kg (i.g.)	Male Balb/c mice	l-Arginine	Enhance SIRT1-mediated deacetylation of p53	Wang et al. (2017)
		30 mg/kg (i.p.)	SD rats	NaT	Increase the interaction of SIRT1 and FOXO1	Rong et al. (2021)
*Others*						
Mogroside IIE	*Siraitia grosvenorii* (Swingle) C. Jeffrey ex A. M. Lu & Zhi Y. Zhang	10 mg/kg (i.p.); 20 µM *in vitro*	Female C57BL/6 mice; AR42J cells/primary acinar cells	Cerulein with LPS	Inhibit IL-9/IL-9R	Xiao et al. (2020)

It is well known that the pathogenesis of AP is closely related to a series of different pathophysiological processes, including self-digestion, inflammatory response, oxidative stress, intracellular Ca^2+^ overload and pancreatic acinar cell necrosis (Xiang et al. 2017). Among them, oxidative stress and inflammatory response are two important aspects in the induction and progression of AP. Oxidative stress is an essential pathogenic factor placed at the crossroad between apoptosis and inflammation. Oxidative stress may lead to the activation of the nuclear factor-κB (NF-κB) and PI3K/AKT signalling pathways, which are involved in the generation and release of inflammatory cytokines in AP (Xiang et al. 2017). Although the development of AP is a complex and highly coordinated process, cellular signal transduction is the main form of intercellular communication. Based on recent studies on the effects of phytochemicals in AP, three major signalling pathways including NF-κB, mitogen-activated protein kinases (MAPKs) and nuclear factor E2-related factor 2 (Nrf2), are of tremendous concern for researchers. Herein, a brief description of the major pathways mentioned above is introduced as follows.

### NF-κB pathway

The transcription factor NF-κB is an important nuclear factor that plays a variety of evolutionarily conserved roles in a plethora of cellular and biological processes. NF-κB affects many cellular responses including inflammation, apoptosis and proliferation by regulating target gene expression. During the development of AP, NF-κB is activated rapidly in pancreatic acinar cells, and then multiple inflammatory cytokines (TNF, IL-6 and IL-1β) and chemokines (MCP-1 and MIP-1α) are significantly increased which can affect vascular permeability and contribute to thrombosis, haemorrhage and tissue necrosis (Steinle et al. 1999). Substantial evidence indicates NF-κB activation to be a key event in the progression of AP (Jakkampudi et al. 2016); therefore, inhibition of NF-κB activation and proinflammatory cytokines release may be a good therapeutic strategy for the attenuation of AP.

### MAPK pathway

The MAPK signalling pathway plays a crucial role in many pathophysiological processes including cell apoptosis, proliferation and differentiation (Ou et al. 2017). MAPKs can specifically phosphorylate serine and threonine residues in response to a variety of stimuli (Kim and Choi 2015). In addition to NF-κB, three major MAPK family members, c-Jun NH2-terminal kinase (JNK), extracellular signal regulated kinase (ERK) and p38 MAPK, are upregulated to alleviate the progression of early AP by reducing pancreatic acinar cells injury and inhibiting inflammation. Notably, the p38 MAPK pathway is also involved during NF-κB activation in the inflammatory cascade. MAPKs can phosphorylate downstream substrates including activated transcription factor 2 (ATF2) and c-Jun, which are closely related to various types of inflammation (Papachristou et al. 2008). Therefore, MAPKs are an important target for the alleviation of AP.

### Nrf2 pathway

Oxidative stress plays a crucial role in the pathogenesis of AP. Reactive oxygen species (ROS) can cause oxidative damage to the lipids and proteins of the cell membranes, resulting in the generation of lipid peroxide byproduct malondialdehyde (MDA) in pancreatic tissue (Folch et al. 1998). Furthermore, ROS can lead to the phosphorylation of p38 MAPK and JNK and the activation of the transcription factor NF-κB, which can further result in the release of inflammatory cytokines in pancreatic acinar cells (Pandol et al. 2010). Nrf2 is a key regulator of a variety of antioxidant enzymes including superoxide dismutase (SOD), glutathione (GSH), NAD(P)H: quinone oxidoreductase 1 (NQO-1) and catalase (CAT). These antioxidant proteins exert cytoprotective effects against pancreatic acinar cells injury. Multiple studies indicate that Nrf2 expression is downregulated in cerulein-induced AP (Liu et al. 2018; Pasari et al. 2019). As an intrinsic defence, the activation of Nrf2/ARE signalling pathways is beneficial for the amelioration of AP.

## The potential mechanisms of phytochemicals in the attenuation of AP

**Figure 1.**
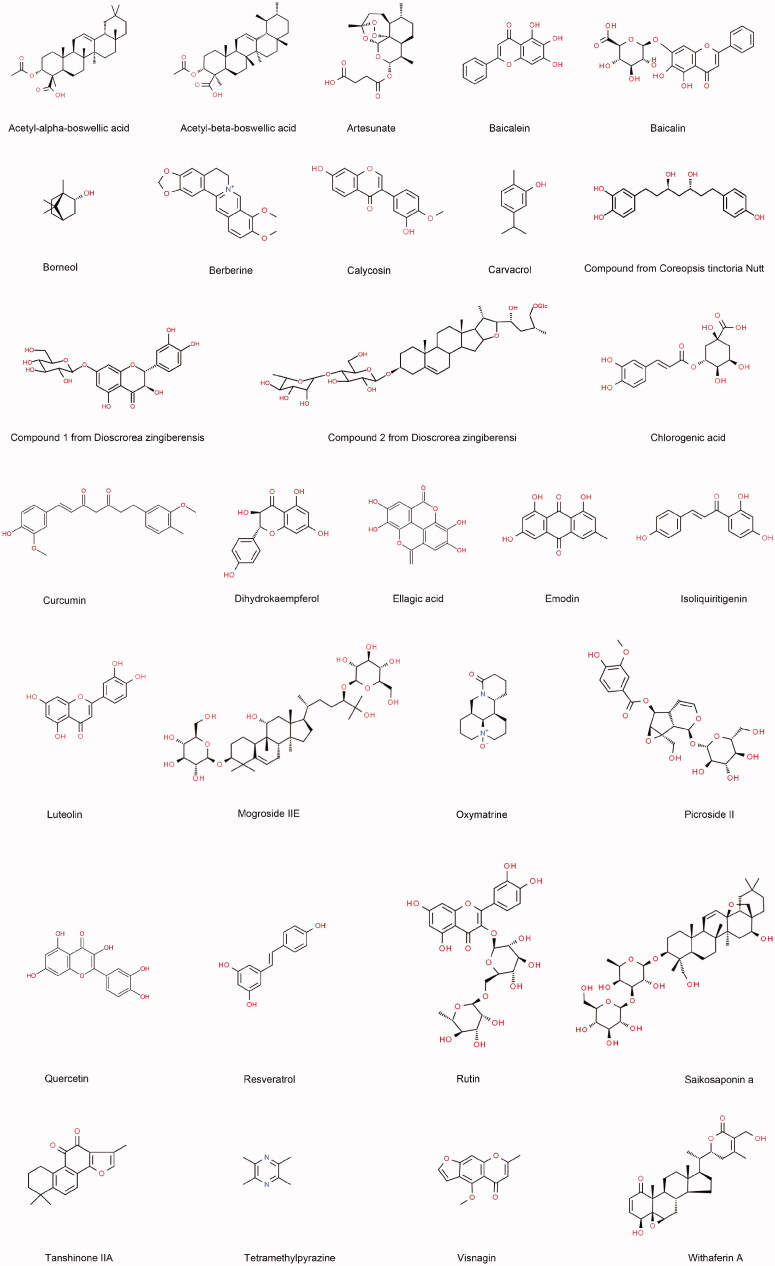
Chemical structures of the phytochemicals that exert protective effects against acute pancreatitis.

**Figure 2.**
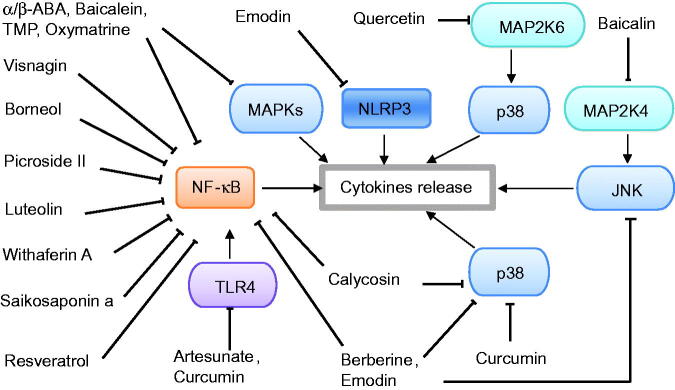
Potential signalling pathways of phytochemicals with anti-inflammatory effects against acute pancreatitis.

**Figure 3.**
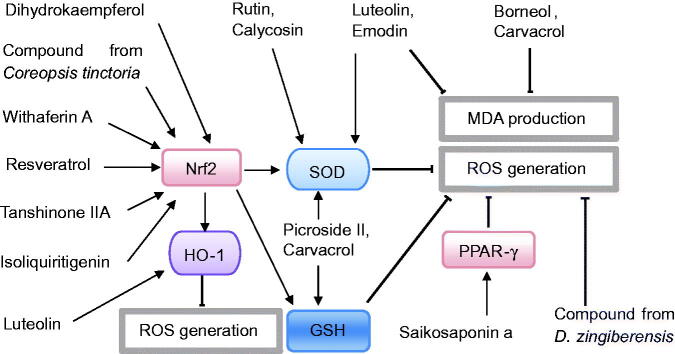
Potential signalling pathways of phytochemicals with antioxidative effects against acute pancreatitis.

Recent *in vivo* or *in vitro* experimental studies published in PubMed and Web of Science between January 2016 and June 2021 were collected using the following search terms: (‘phytochemicals’ OR ‘medicinal plant’ OR ‘traditional medicine’) AND (‘pancreatitis’ OR ‘pancreatic damage’ OR ‘pancreatic injury’). A total of 30 phytochemicals with potential therapeutic effects were reviewed and summarized systematically. According to the modes of these phytochemicals in experimental pancreatitis, the phytochemicals are mainly classified into four categories: phytochemicals with anti-inflammation, phytochemicals with antioxidation, phytochemicals with anti-inflammation and antioxidation, and others. The chemical structures and signalling pathways of the phytochemicals in AP are shown in Figures 1–3. Then, the underlying mechanisms of each phytochemical are illustrated in detail.

### Phytochemicals with anti-inflammatory effects

#### Acetyl-α/β-boswellic acid

Acetyl-α/β-boswellic acid (α/β-ABA), the active ingredients isolated from *Boswellia sacra* Flückiger-Dupiron (Burseraceae), protected against pancreatitis by downregulating MAPK family phosphorylated proteins including phosphorylated p38 MAPK, ERK1/2 and JNK. Furthermore, it reduced phosphorylated c-Jun in the nucleus and then decreased inflammatory cytokines, such as nitric oxide (NO), TNF-α and interleukins (IL-6, IL-10 and IL-1β), both *in vivo* and *in vitro*. Finally, α/β-ABA reversed the serum elevation of amylase levels and alleviated pancreatic oedema and inflammatory cell infiltration in pancreatic tissue. This study indicates that α/β-ABA may be pursued for drug development against AP (Zhang et al. 2020).

#### Artesunate

Artesunate is a water-soluble hemisuccinate derivative of artemisinin which is an active ingredient isolated from the traditional Chinese herb *Artemisia annua* L. (Asteraceae). It has diverse pharmacological properties including anti-inflammatory action, antibacterial activity and antitumor effects (Li et al. 2012). Artesunate significantly increased the survival of severe acute pancreatitis (SAP) rats, improved pancreatic pathology while decreasing serum amylase, lipase activities and pancreatic IL-6 and IL-1β levels. Another study demonstrated that the protective effect of artesunate against pancreatic injury was associated with the inhibition of the Toll-like receptor 4 (TLR4)/NF-κB signalling pathway, thus reducing the release of proinflammatory cytokines (Cen et al. 2016).

#### Baicalein

Baicalein (5,6,7-trihydroxy-2phenyl-4H-1-benzopyran-4-one) is a major active compound extracted from the root of *Scutellaria baicalensis* Georgi (Lamiaceae). It has been shown to exert significant antioxidant and anti-inflammatory activities (Pu et al. 2019). Baicalein has been reported to inhibit the production of inflammatory mediators, such as TNF-α, IL-6, interferon-γ and macrophage chemoattractant protein-1 (MCP-1) (Krakauer et al. 2001; Shen et al. 2003). Baicalein significantly suppressed the inflammatory response and attenuated pathological damage in rats (Li et al. 2015). It improved survival in rats with SAP greatly. Baicalein exerted its anti-inflammatory effects by inhibiting the expression of NF-κB p65 and the phosphorylation of p38 MAPK, ERK and signal transducer and activator of transcription 3 (STAT3) (Pu et al. 2019).

#### Baicalin

Baicalin (5,6,7-trihydroxy-2-phenyl-4H-1-benzopyran-4-one 5,6-dihydroxy-4-oxo-2phenyl-4H-1-benzopyran-7-yl-β-d-glucopyranosiduronic acid, C_21_H_18_O_11_) is a glucuronide of baicalein (Zhen et al. 2021). Baicalin has various pharmacological effects, including anti-inflammatory and anticancer effects (Ming et al. 2018). Baicalin can exert protective effects against AP by increasing miR-15a level and inhibiting CDC42 (cell division cycle 42)/MAP3K1 (mitogen-activated protein kinase kinase kinase 1), which can affect AP as a brake by targeting mitogen-activated protein kinase kinase 4 (MAP2K4) and inhibiting the JNK signalling pathway (Zhen et al. 2021). It can also suppress the inflammatory response by downregulating NF-κB and protein kinase D1 (PKD1) expression (Qian et al. 2018).

#### Berberine

Berberine is a major isoquinoline alkaloid isolated from *Coptis chinensis* Franch (Ranunculaceae), barberry and Oregon grape. It has been used extensively to treat diarrhoea, gastroenteritis and various infectious disorders in traditional Chinese medicine (Choi et al. 2017). Furthermore, berberine decreased the levels of serum amylase and lipase, the levels of proinflammatory mediators (such as TNF-a, IL-1β and IL-6), the levels of inducible nitric oxide synthase (iNOS) and myeloperoxidase (MPO) activity in AP mice. Finally, berberine inhibited AP through suppression of the activation of the JNK, NF-κB and p38 MAPK signalling pathways (Choi et al. 2016, 2017).

#### Chlorogenic acid

Chlorogenic acid (CGA), an important biologically active polyphenol, was first identified in *Lonicera japonica* Thunb. (Caprifoliaceae). It is abundant in a variety of plants including green coffee beans, cocoa leaves and seeds, vegetables and fruits (Tajik et al. 2017; Tarasiuk and Fichna 2019). Chlorogenic acid reduced the histological severity of pancreatitis and the levels of pancreatic lipase and amylase. In addition, macrophage migration inhibitory factor (MIF) was critical in mediating inflammatory cytokines and it was associated with the severity of AP. Chlorogenic acid significantly downregulated the levels of inflammatory cytokines and chemokines, such as IL-6, MIP-2 and MIF, in mouse serum and pancreatic tissue. Chlorogenic acid exerted an anti-inflammatory effect on l-arginine-induced AP (Ohkawara et al. 2017).

#### Oxymatrine

Oxymatrine is an alkaloid derived from the Chinese herb *Sophora alopecuroides* L. (Fabaceae), with immunosuppression and anti-inflammatory potential. Oxymatrine protected against l-arginine-induced pancreatic damage *in vitro* and *in vivo* by regulating proinflammatory cytokines (TNF-α, IL-6, IL-1β, IL-17A/IL-17F and IFN-γ) and the MAPK/NF-κB signalling pathway (Zhang Z et al. 2019).

#### Quercetin

Quercetin (3,3′,4′,5,7-pentahydroxyflavone), a type of flavone compound that is ubiquitously distributed in fruits and vegetables, exhibits various biological functions. Its anti-inflammatory ability appears to be worthy of particular attention. Quercetin exerted a protective effect against pancreatic cell injury by suppressing p38 MAPK signalling via upregulation of miR-216b and reduction of TNF-α expression. In addition, long noncoding RNA nuclear-enriched abundant transcript 1 (NEAT1) was also involved in the regulation of mitogen-activated protein kinase kinase 6 (MAP2K6) via miR-216b and could be suppressed by quercetin. Quercetin also attenuated the levels of inflammatory mediators (TNF-α, IL-10 and IL-6) via the suppression of TNF receptor-associated factor 2 (TRAF2) and mitogen-activated protein kinase kinase kinase 5 (MAP3K5) located upstream of the MAPK signalling pathway (Sheng et al. 2021).

#### Saikosaponin

Saikosaponins (SSs), including saikosaponin a (SSa), are the major bioactive ingredients extracted from Radix Bupleuri. Radix Bupleuri is derived from the dried roots of *Bupleurum chinense* DC. (Apiaceae). SSs possess diverse pharmacological properties including antioxidant, anti‑inflammatory and immune regulatory effects. Among the SSs, SSa is greatly attractive due to its significant anti‑inflammatory activity (Du ZA et al. 2018). A recent study showed that administration of SSa attenuated hyperlipidaemic pancreatitis in rats by improving lipid metabolism effectively characterized by a decrease in total cholesterol and triglycerides in serum. In addition, it significantly decreased the levels of pancreatic enzymes (MPO, amylase and lipase) and reduced proinflammatory cytokines (TNF‑α, IL-1β and IL-6) release by suppressing the NF-κB signalling pathway and promoting the expression of peroxisome proliferator-activated receptor-γ (PPAR-γ) (Feng et al. 2019). Taken together, SSa exerted therapeutic effects on hyperlipidaemic pancreatitis.

#### Tetramethylpyrazine/ligustrazine

Tetramethylpyrazine (TMP), also known as ligustrazine, is one of the major active constituents of *Oreocome striata* (Wall. ex DC.) Pimenov & Kljuykov (Apiaceae). TMP has long been used for treating inflammatory and cardiovascular diseases clinically in China due to its anti-inflammatory and vessel protection effects. TMP exerted a protective effect against pancreatitis by inhibiting NF-κB activation followed by a decrease in the levels of inflammatory factors; it also induced pancreatic cell apoptosis by enhancing the expression of cleaved caspase-3 and p53, reducing Bcl-2 expression (Chen J et al. 2016; Chen L et al. 2019). In addition, ligustrazine alleviated AP by accelerating acinar cell apoptosis at an early phase via suppression of the p38 MAPK and ERK pathways. Moreover, ligustrazine downregulated the levels of inflammatory factors (TNF-α, IL-1β and IL-6) and the activities of amylase and MPO (Chen et al. 2016). In summary, TMP/ligustrazine has been demonstrated to attenuate injury to the pancreas and represents a potential therapeutic strategy in the management of patients with AP.

#### Visnagin

Visnagin is an antioxidant furanocoumarin derivative from the biennial herb *Ammi visnaga* (L.) Lam. (Apiaceae). It has been demonstrated that visnagin is advantageous for the patients with urolithiasis and hypertriglyceridaemia. It not only has beneficial antioxidant effects, but also has anti-inflammatory effects by reducing the production of proinflammatory cytokines (Lee et al. 2010). Visnagin enhanced antioxidant defence by improving Nrf2 expression accompanied by alterations in the levels of MDA, GSH, nitrite and MPO. Moreover, it also inhibited pancreatic inflammation by suppressing NF-κB activation and reduced the levels of IL-1β, IL-6, TNF-α and IL-17 in a dose-dependent manner (Pasari et al. 2019).

#### Withaferin A

Withaferin A (WFA), often used in the Ayurvedic medicine system of Indian, is obtained from the roots of *Withania somnifera* (L.) Dun. (Solanaceae) (nightshades). It was proven to be effective in many acute inflammatory disorders due to its anti-inflammatory activity. In the cerulein-induced AP mouse model, WFA protected the structure of the pancreas by reducing massive oedema and inflammation in a dose-dependent manner. Moreover, WFA increased the levels of pancreatic MPO, and resulted in the nuclear translocation of Nrf2. In addition, WFA restored the increased levels of NF-κB and upregulated the expression of Bcl-2 in cerulein-induced AP. Taken all together, WFA can be a good choice for reducing the severity of AP (Tiruveedi et al. 2018).

### Phytochemicals with antioxidative effects

#### Carvacrol

Carvacrol, a predominant monoterpenoid obtained from the plant *Origanum vulgare* Linn. (Lamiaceae), is recognized as a traditional medicine in some countries (Anchi et al. 2017). Carvacrol, with good antioxidant activity, provides protection against lipid peroxidation (Quiroga et al. 2015). Carvacrol significantly decreased the pancreas histopathological scores and serum levels of amylase and lipase in cerulein-induced AP by modulating endogenous antioxidant defence activities marked by elevation in SOD, CAT and glutathione peroxidase (GSH-Px) activities (Kilic et al. 2016).

#### Dihydrokaempferol

Dihydrokaempferol (DHK) is a natural flavonoid extracted from the medicinal plant *Bauhinia championii* (Benth.) Benth. (Fabaceae). It has multiple pharmacological functions including anti-inflammation and antioxidation. A recent study demonstrated that DHK exerted an antioxidative effect on AP by reducing the level of Keap1 and promoting transcriptional activation of Nrf2, which can increase the expression of downstream antioxidants and detoxification enzymes, thus suppressing ROS generation. DHK can also improve cell necrosis induced by AP (Liang et al. 2020).

#### Isoliquiritigenin

Isoliquiritigenin (ISL) is a natural compound derived from various edible plants, e.g., soybeans (*Glycine max* (L.) Merr. (Fabaceae)), licorice (*Glycyrrhiza glabra* L. (Fabaceae)), and shallots (*Allium ascalonicum* L. (Alliaceae)). It has been used for several years as a traditional Chinese medicine in clinical practice. ISL has multiple pharmacological functions including antioxidative, anti-inflammatory and antitumor activities (Jin et al. 2016). In the cerulein-induced AP mouse model, ISL dose-dependently alleviated the histopathological change in pancreatic tissue. Moreover, ISL suppressed oxidative stress by activating the Nrf2/HO-1 (heme oxygenase-1) pathway and increasing antioxidative enzyme (SOD and GSH) levels. These data suggested that ISL may be a promising therapeutic chemical for AP in the future (Liu et al. 2018).

#### Rutin

Rutin (quercetin-3-rhamnoglucoside), a flavonoid derived from several citric fruits and grapes, has been widely explored due to its various pharmacological properties including antioxidant, anti-inflammatory and antinociceptive activities (Abreu et al. 2016). In an l-arginine-induced AP model, rutin attenuated histopathological damage and levels of serum amylase; rutin diminished oxidative stress and hindered lipid peroxidation (Abreu et al. 2016). This study provided complementary evidence that rutin exerted potent antioxidant activity and reduced pancreatic inflammatory responses. These findings indicate that rutin might be an alternative therapeutic agent against AP.

#### Tanshinone IIA

Tanshinone IIA (TSA) is a liposoluble compound isolated from *Salvia miltiorrhiza* Bunge (Lamiaceae). It is effective at treating various disorders including tumour progression, cardiovascular disease and liver disease. In addition, TSA has various pharmacological properties including antiangiogenic, anti-inflammatory and antioxidant activities (Ren et al. 2019; Shi et al. 2019). TSA reduced the levels of serum amylase and lipase and ameliorated the histopathological damage induced by cerulein in the pancreas. In addition, TSA significantly inhibited oxidative stress, protected the structure of the endoplasmic reticulum and mitochondria, and promoted activation of the Nrf2/HO-1 pathway in pancreatic tissue (Chen et al. 2020). These results indicated that TSA is a promising therapeutic drug for AP, and more research should be performed in the future.

#### (2R,3R)-Taxifolin 7-O-β-d-glucopyranoside

*Coreopsis tinctoria* Nutt. (Asteraceae), an annual herb, is a popular traditional medicine in Asia and North America that has been widely used in the treatment of several diseases including pain, diabetes mellitus and diarrhoea (Du D et al. 2018). (2*R*,3*R*)-taxifolin 7-*o*-β-d-glucopyranoside (C1) was initially isolated and identified from *Coreopsis tinctoria* (Du D et al. 2018). The study showed that it exerted protective effects against acute pancreatic injury through its antioxidant properties. Flavonoid C1 effectively activated the Nrf2/HO-1 signalling pathway and elevated the levels of SOD and GSH in pancreatic tissue. Flavonoid C1 significantly decreased ROS production and ATP depletion at least partly by upregulating the Nrf2/ARE-mediated antioxidant pathway (Du D et al. 2018). These results indicate the potential application of C. tinctoria to treat AP.

#### (3R,5R)-3,5-Dihydroxy-1-(3,4-dihydroxyphenyl)-7-(4-hydroxyphenyl) heptane

Steroidal saponins isolated from *Dioscorea zingiberensis* C. H. Wright (Dioscoreaceae) are well known. The rhizome of *Dioscorea zingiberensis*, also referred to ‘Huang Jiang’, has been used to treat chronic lung disease, coronary heart ailments, bacterial infection and various other maladies. They have multiple pharmacological activities, such as anticancer, anti-inflammatory, antioxidant and neuroprotective activities. (3*R*,5*R*)-3,5-dihydroxy-1-(3,4-dihydroxyphenyl)-7-(4-hydroxyphenyl) heptane was isolated and identified from *Dioscorea zingiberensis*. It has been demonstrated that this compound can be a potential candidate for mediating mitochondrial dysfunction. According to the findings reported by Du et al. (2017), this compound protected against pancreatic acinar cell injury by reducing ATP depletion and excessive ROS production. Since it is a newly extracted compound, further studies are necessary to validate its therapeutic potential.

#### 26-O-β-d-Glucopyranosyl-3β,22α,26-trihydroxy-25(R)-furosta-5-en-3-O-[α-l-rhamnopyranosyl-(1→4)]-β-d-glucopyranoside

26-o-β-d-Glucopyranosyl-3β,22α,26-trihydroxy-25(R)-furosta-5-en-3-o-[α-l-rhamnopyranosyl-(1→4)]-β-d-glucopyranoside is another compound of diosgenyl saponins isolated from *Dioscorea zingiberensis* C. H. Wright (Dioscoreaceae). This saponin compound could inhibit the activation of the necrotic cell death pathway in a concentration-dependent manner, and is also capable of preventing the loss of ATP production, mitochondrial membrane potential and ROS generation in pancreatic acinar cells. Moreover, it also significantly reduced histological pancreatic injury in mice (Zhang et al. 2016). Remarkably, the potential mechanism of this compound is still unclear, and much more research is still needed.

### Phytochemicals with anti-inflammatory and antioxidative effects

#### Borneol

Borneol is a natural bicyclic monoterpenoid alcohol-based aromatic flavouring substance isolated from the perennial herb *Acorus calamus* L. (Acoraceae). Borneol exhibits different biological effects by inhibiting oxidative stress, apoptosis and inflammatory reactions (Yu et al. 2016). Borneol not only reduced the levels of MDA and nitrite, but also elevated the levels of reduced GSH in the mouse pancreas by significantly enhancing nuclear factor Nrf2 and SOD1 expression. In addition, borneol substantially inhibited the levels of proinflammatory cytokines (TNF-α, IL-1β and IL-6) and iNOS by reducing phospho-NF-κB p65 expression. In summary, borneol is beneficial for the amelioration of AP, potentially, by reducing oxidative damage and inflammation via the regulation of the Nrf2/NF-κB pathway (Bansod et al. 2021).

#### Calycosin

Calycosin, an isoflavone isolated from *Astragalus membranaceus* (Fisch.) Bunge (Fabaceae) with antioxidant and anti-inflammatory activities, is widely used in traditional Chinese medicines. Calycosin has been demonstrated to exhibit various biological activities, including anti-inflammatory (Su et al. 2016) and neuroprotective (Wang et al. 2018) properties. In cerulein-induced AP mice, calycosin treatment protected pancreatic tissue from oxidative damage by reducing MPO activity and increasing SOD activity. In addition, calycosin exerted an anti-inflammatory effect against pancreatic damage by mitigating the levels of the inflammatory cytokines TNF-α, IL-1β and IL-6. Calycosin decreased the expression level of NF-κB p65 and inhibited the phosphorylation levels of IκBα and p38 MAPK (Ma et al. 2018). Therefore, calycosin with great potential may be recognized as a therapeutic drug for AP.

#### Curcumin

Curcumin, a natural polyphenolic compound extracted from the perennial herb *Curcuma longa* L. (Zingiberaceae), exhibits a wide range of biological activities (Siriviriyakul et al. 2019; Tarasiuk and Fichna 2019). Curcumin significantly improved histopathological changes, including pancreatic inflammation, oedema and fat necrosis; it caused a decrease in the number of MPO-positive cells, NF-κB-positive cells, TUNEL-positive cells and the expression of the oxidative stress marker 4-hydroxynonenal (Siriviriyakul et al. 2019). Moreover, curcumin reduced the serum levels of TNF‑α and C-reactive protein (CRP), decreased amylase activity, and alleviated the severity of AP by inhibiting the p38 MAPK and TLR-4/NF-κB signalling pathway (Zhong 2015; Wang et al. 2019). In addition, curcumin significantly decreased pancreatic MDA and iNOS, while increasing the total antioxidant capacity (Ragy et al. 2019). Taken all together, curcumin could attenuate acute pancreatic damage via antioxidant, anti-inflammatory and anti-apoptotic effects (Siriviriyakul et al. 2019).

#### Ellagic acid

Ellagic acid, a natural antioxidant derived from phenolic acids, is widely found in many fruits and vegetables. Ellagic acid is well known to reduce lipid peroxidation and protect against oxidative stress-cell injury. Furthermore, it has a variety of properties including anti-inflammatory, antifibrotic, antiangiogenic and antiproliferative effects. A recent study showed that ellagic acid not only greatly increased the total antioxidant capacity, but also reduced the serum levels of the inflammatory cytokines TNF-α, IL-1β and IL-6. The protective effects of ellagic acid on pancreatic damage against l-arginine administration were further confirmed by histopathological and biochemical evaluations of pancreatic tissue (Yılmaz et al. 2016).

#### Emodin

Emodin (1,3,8-trihydroxy-6-methyl-anthraquinone) is an anthraquinone isolated from the traditional Chinese herb of *Rheum rhabarbarum* L. (Polygonaceae). Emodin is also a major active ingredient of Qingyi decoction that has been clinically used to treat AP over the past 30 years (Xia et al. 2012). Emodin significantly attenuated pancreatic histopathological damage, pancreatic MPO activity, iNOS content and the levels of amylase and lipase in pancreatic tissues. Moreover, emodin suppressed the P2X7/NLRP3 pathway and NLRP3 inflammasome activation followed by the reduction of pro‑inflammatory factors including IL‑1β and IL‑18, which was beneficial for the recovery of pancreatitis (Ning et al. 2017; Zhang Q et al. 2019). Another *in vitro* study demonstrated that emodin reduced the trypsin content and lipase activity, increased the levels of matrix metalloproteinase (MMP), and inhibited the generation of ROS and mitochondrial damage. Furthermore, emodin significantly downregulated ROS-mediated activation of the JNK and p38 MAPK pathways. Emodin also ameliorated pancreatic cell injury by reducing the release of the inflammatory mediators IL-6 and TNF-α (Zhao JY et al. 2019). Emodin attenuated serum amylase levels and histological assessment of oedema, vacuolization, inflammation and necrosis; it also significantly inhibited nuclear NF‑κB DNA‑binding activity followed by a decrease in TNF‑α, IL‑6 and IL‑1β. It is worth mentioning that this protective effect of emodin was potentially associated with the reduction of MDA and the elevation of SOD in pancreatic tissue (Yao et al. 2015).

#### Luteolin

Luteolin (3,4′,5′,7-tetrahydroxyflavone), a natural flavonoid, was first isolated from the annual herb *Reseda odorata* L. (Resedaceae). Currently, it has been found in many herbal medicines and vegetables. Luteolin has long been used for the treatment of diseases related to inflammation and oxidative stress, such as acute myocardial infarction and acute lung injury. It was proven that luteolin not only significantly improved HO-1-mediated antioxidant activity, but also ameliorated the inflammatory response by inhibiting the translocation of NF-κB in the mouse pancreas. Furthermore, luteolin greatly reversed pathological changes and reduced the levels of TNF-α and IL-6 in the pancreas. Luteolin can be a potential protective agent against SAP (Xiong et al. 2017).

#### Picroside II

Picroside II (h-d-glucopyranoside,1a,1b,2,5a,6,6a-hexahydro-6-[(4-hydroxy-3-methoxybenzoyl) oxy]-1a(hydroxymethyl) oxirenocyclopenta [1,2-c] pyran2-yl), extracted from *Picrorhiza scrophulariiflora* Pennell (Plantaginaceae), has anti-inflammatory and antioxidant activities. Picroside II suppressed the autophagic activity of SAP via the inhibition of TNF-α, NF-κB and SIRT 1 (sirtuin 1)-deacetylated light chain 3 (LC3, an indicator of autophagic activity). In addition, picroside II not only significantly increased the levels of SOD and GSH, but also reduced the levels of TNF-α, IL-1β and IL-6. Collectively, picroside II exerted a protective effect against SAP via NF-κB-dependent autophagy (Piao et al. 2017).

#### Resveratrol

Resveratrol (3,5,4′-trihydroxy-*trans*-stilbene), a natural polyphenol-rich ingredient extracted from the herb *Polygonum cuspidatum* Siebold & Zucc. (Polygonaceae), can exert antioxidant, chemopreventive and anti-inflammatory effects. Due to its antioxidative and immunomodulatory properties, resveratrol exerts its beneficial effects in the management of AP through modulation of many cytokines, transcriptional elements and microRNAs (Agah et al. 2021). Resveratrol can not only maintain the structural integrity of cellular membranes by reducing lipid peroxidation, but also improve the antioxidant defence system via the activation of the Nrf2/ARE pathway. Resveratrol can inhibit protein carbonylation via the increased expression and activity of SIRT1 (Agah et al. 2021). Furthermore, it can increase the interaction of SIRT1 and forkhead box protein O1 (FOXO1) with substantial decrease in acetyl-FOXO1 expression (Rong et al. 2021). In addition, resveratrol can inhibit the activation of NF-κB, PI3Ks/AKT, activator protein 1 (AP-1) and TNF-α, which are responsible for various inflammatory processes (Tsang et al. 2016). Resveratrol can inhibit leukocyte adhesion to pancreatic cells via the suppression of intercellular cell adhesion molecule (ICAM) and vascular cell adhesion molecule (VCAM) (Agah et al. 2021). Resveratrol can enhance SIRT1-mediated deacetylation of p53, heat shock factor 1 (HSF1) and IL-10 (Wang et al. 2017). Resveratrol can suppress calcium overload, decrease trypsinogen activation and improve mitochondrial and microcirculatory function (Agah et al. 2021). Taken together, resveratrol might be an effective therapeutic component for the treatment of AP.

### Others

#### Mogroside IIE

Mogroside IIE, a major component isolated from unripe *Siraitia grosvenorii* (Swingle) C. Jeffrey ex A. M. Lu & Zhi Y. Zhang (Cucurbitaceae), has long been used to inhibit inflammation. It is known that intrapancreatic trypsinogen activation is essential in the pathogenesis of AP (Saluja et al. 2019). Herein, the inhibition of trypsinogen activation would be beneficial for the treatment of AP. Recent evidence demonstrated that mogroside IIE ameliorated AP through inhibition of the IL-9/IL-9 receptor pathway, which increased cytosolic calcium and the activity of cathepsin B and trypsin, and induced impaired autophagy (Xiao et al. 2020). Interestingly, mogroside IIE reduced the levels of lipase and amylase in serum without influencing inflammation significantly. Taken together, mogroside IIE ameliorated pancreatic injury, enzyme activity and cytokines release in AP.

## Conclusions and perspective

Traditional Chinese medicine decoction has long been used to treat AP as an additional or alternative therapeutic strategy. Decoctions are effective in reducing the mortality rate in patients with SAP (Li et al. 2017). They can greatly improve gastrointestinal motility and inhibit inflammatory response in AP. It is worth noting that phytochemicals with multiple protective abilities can improve AP pathogenesis and attenuate pancreatic cell injury. Herein, the phytochemicals that can exert beneficial effects on acute pancreatic injury are systematically summarized in this paper according to recent studies.

The event that local inflammation escalates into systemic inflammation is a key determinant of the severity of AP. Inflammatory stimuli can activate many intracellular signalling pathways, including NF-κB pathway and three MAPKs (p38, ERK and JNK) pathways (Awasthi et al. 2021). The MAPK pathway can convert extracellular signals into a multitude of cellular responses. MAPK can phosphorylate a large number of substrates and induce NF-κB activation, contributing to cytokine production (Chan et al. 2001). Moreover, inhibition of p38 MAPK resulted in a significant inhibition of NF-κB by reducing NF-κB-dependent transcription but did not affect NF-κB translocation or DNA binding (Beyaert et al. 1996; Catley et al. 2004). On the other hand, abrogation of NF-κB signalling resulted in sustained p38 MAPK activation (Langereis et al. 2010). In addition, inhibition of either JNK or ERK1/2 attenuated TNF-α-induced NF-κB activation (Olejarz et al. 2014). Therefore, NF-κB and MAPK are vital anti-inflammatory targets for AP. Hence, the regulation of the immune system and inflammatory mediators is an important area of much interest from the point of both drug discovery and therapeutic intervention. Recently, natural inhibitors with minimal side effects targeting NF-κB and MAPK have been discovered and characterized. As shown in Table 1 and Figure 2, artesunate, SSa, baicalein, berberine, curcumin, oxymatrine and TMP suppressed the inflammatory response in the pancreas and reduced the release of inflammatory mediators including TNF-α, IL-1β and IL-6 by inhibiting the NF-κB pathway; while α/β-ABA, baicalein, baicalin, quercetin, berberine, curcumin, oxymatrine, emodin and TMP decreased inflammatory cytokines and alleviated pancreatic oedema and inflammatory cell infiltration in pancreatic tissue by downregulating MAPKs (ERK, p38 and JNK) phosphorylated proteins. Interestingly, baicalein, curcumin, emodin, oxymatrine and TMP can regulate both NF-κB and MAPK pathways which are of great importance in the pathogenesis of AP.

In addition to inflammation, oxidative stress has been implicated in the pathogenesis of AP. The transcription factor Nrf2 plays a vital role in the protection of cells against oxidative stress. As shown in Table 1 and Figure 3, phytochemicals including DHK, TSA and ISL, exerted antioxidative effects against pancreatic injury by activating the Nrf2 pathway and suppressing ROS generation. Importantly, phytochemicals (such as emodin, baicalein, luteolin and resveratrol) not only inhibited oxidative stress by activating the Nrf2 pathway, but also suppressed inflammation and reduced cytokine release by downregulating the NF-κB or MAPK pathways. Finally, phytochemicals with anti-inflammatory and antioxidant abilities may be promising natural product candidates for the attenuation of AP according to these experimental studies.

Although much evidence has demonstrated that these phytochemicals exert therapeutic effects against AP in experimental animal models, whether these phytochemicals are beneficial for the treatment of AP in humans is still unclear. In 2015, one single-institution, double-blind, randomized, placebo-controlled drug trial to determine the safety and efficacy of pentoxifylline (an analogue of theobromine extracted from cocoa beans) in patients with predicted severe AP was reported by Vege et al. (2015). Although there were no differences between the pentoxifylline group and placebo group in terms of the inflammatory markers and the change in SIRS score and APACHE II, the pentoxifylline group with no adverse effects had fewer intensive care unit (ICU) admissions and shorter ICU and hospital stays. Due to the small sample size of this clinical study, a larger-scale study of pentoxifylline in AP is needed to confirm its efficacy. In 2016, another multicentre, single-blinded, randomized controlled trial (no. NCT02947932, phase 4) was registered, with the purpose that oral resveratrol before endoscopic retrograde cholangiopancreatography (ERCP) could reduce overall pancreatitis in patients undergoing ERCP. Unfortunately, the results of this clinical study have not been reported as of late 2021.

Collectively, more preclinical investigations including pharmacodynamic, pharmacokinetic and toxicological data are still needed to ensure the safety and to confirm efficacy of these compounds in further clinical applications. Meanwhile, the research for compounds with even more potential, that are naturally occurring in plants should be continued. Herein, this review intends to highlight the current understanding of phytochemicals in the protection of AP.
